# Low intensity pulsed ultrasound (LIPUS) use for the management of instrumented, infected, and fragility non-unions: a systematic review and meta-analysis of healing proportions

**DOI:** 10.1186/s12891-021-04322-5

**Published:** 2021-06-11

**Authors:** Ross Leighton, Mark Phillips, Mohit Bhandari, Robert Zura

**Affiliations:** 1 Division of Orthopaedic Surgery, Dalhousie University, Halifax, Nova Scotia Canada; 2 Department of Health Research Methods, Evidence, and Impact, McMaster University, Hamilton, Ontario Canada; 3 Division of Orthopaedic Surgery, McMaster University, Hamilton, Ontario Canada; 4 Department of Orthopaedic Surgery, Louisiana State University Health Sciences Center, New Orleans, Louisiana USA

## Abstract

**Background:**

Non-union occurs in approximately 5 to 10% of fracture patients, with certain bones at greater risk of failing to heal. Non-unions have a significant impact on socioeconomic costs and the patients short and long-term quality of life. Low intensity pulsed ultrasound (LIPUS) is a non-invasive therapy for non-union treatment that can improve the long-term outcome. The purpose of this study is to summarize the available literature assessing LIPUS potential to improve the union rate in instrumented, infected, and fragility non-unions.

**Methods:**

A literature search was conducted in the MEDLINE, EMBASE, and CINAHL databases for all relevant literature on the healing rates of LIPUS utilized in instrumented, infected, and fragility non-unions. Study characteristics were summarized for each of the included studies. The percentage of healed patients (healing rate), for instrumented, infected, and fragility fracture non-union patients were pooled from each included study.

**Results:**

The literature search identified a total of 326 articles, while searching reference lists and grey literature identified an additional 3 articles. There was a total of 29 articles included in this review, with 20 articles included within the quantitative synthesis of healing rates. The most common design of included studies was case series (17 articles), followed by case reports (9 articles). Studies were primarily retrospective (18 studies), with an additional 10 prospective studies. Non-union healing rates were 82% (95% CI: 76 to 87%) in instrumented, 82% (95% CI: 70 to 95%) in infected, and 91% (95% CI: 87 to 95%) in fragility fracture patients with non-unions.

**Conclusion:**

This study has provided a thorough overview of the current literature on LIPUS treatment for instrumented, infected, and fragility fracture non-unions. The healing rates for non-unions in these subgroups were comparable to healing rates observed with LIPUS use in general non-union literature. LIPUS treatment should be considered as a conservative non-surgical treatment option to potentially reduce the socioeconomic impact and improve the quality of life of these unfortunate patients.

**Level of evidence:**

4 (systematic review of primarily case series data)

**Supplementary Information:**

The online version contains supplementary material available at 10.1186/s12891-021-04322-5.

## Background

Non-union occurs in approximately 5 to 10% of fracture patients, with certain bones at greater risk of failing to heal [1, 2]. There are a number of comorbidities that increase the risk of non-union, including smoking, alcoholism, obesity, osteoarthritis, rheumatoid arthritis, and type 2 diabetes [1, 3]. Previous use of certain medications, such as opioids or anticoagulants or smoking can also increase the risk of non-union [1]. Ultimately, the risk of non-union is a multifaceted combination of fracture severity, location, comorbidities, and other medication use [1].

Non-unions have a significant impact on the quality of life of patients due to a need for further interventions, which is often additional surgery. Surgical management of non-unions also creates a large socioeconomic impact for both patients and the healthcare system [4, 5]. Estimates suggest that the cost of non-union management is $25,556 per patient, with increased need for opioids, and substantial increased use of the health care system [5]. Non-unions that occur in instrumented, fragility fractures, or infected fractures can be particularly challenging to manage [6, 7]. These non-union subgroups are associated with increased risks of further complications, which can increase the socioeconomic impact to the patient and their families. Avoiding the need for additional surgery through conservative management of non-unions can have a substantial benefit to patients and the healthcare system alike, if the nonunion can be resolved nonoperatively.

Low intensity pulsed ultrasound (LIPUS) is a well-recognized non-invasive therapy for non-union treatment. This therapy provides a low-intensity ultrasound signal to the fracture site [6]. LIPUS’s original FDA approved indications were for healing non-unions (excluding skull and vertebrae) and accelerating time to healing of fresh fractures of the tibia and radius [6].. Recently, LIPUS (EXOGEN Ultrasound Bone Healing System, Bioventus, Durham North Carolina) indications have been updated to include treatment of fragility fractures, instrumented fractures, and infected non-unions. Due to these updated indications, it is important to provide the available data from the literature to support LIPUS use in these particularly problematic non-union cases. The purpose of this study is to summarize the available literature assessing LIPUS (EXOGEN) heal rates in instrumented, infected, and fragility fracture non-unions.

## Methods

### Literature search

This review followed the PRISMA guidance for systematic review reporting [8]. A literature search was conducted in the MEDLINE, EMBASE, and CINAHL databases with no limits on the year of publication (Additional file 1). The reference lists of included studies were also hand-searched to identify any eligible studies that the search did not capture. Only articles published in English were included.

### Study selection

Studies were screened in duplicate at the title/abstract and full text stage. All articles that were included by at least one reviewer at the title/abstract stage proceeded to full text review. Any disagreements in eligibility at the full text stage were resolved via consensus meetings. Studies that met the following inclusion criteria were eligible: 1) Use of LIPUS (EXOGEN) for non-union/delayed union fracture management, 2) Healing rate is reported as an outcome, 3) Healing rates must be reported for at least one of the risk groups of; fragility fracture non-unions, instrumented non-unions, or infected non-unions, and 4) Primary investigations on humans. The following exclusion criteria were used:1) Use of LIPUS for conditions other than non-unions, 2) Use of non-LIPUS bone growth stimulators, 3) Use of LIPUS in fresh fractures, 4) Use of LIPUS in conjunction with other non-union treatments, 5) Systematic reviews, literature reviews, or evidence synthesis publications, as these are not primary investigations, and 6) Animal studies.

### Data extraction

All data was extracted using a standardized data extraction form. Study characteristic data on the country, study design, prospective/retrospective enrollment, number of participants, mean age, percentage male and female, and bones treated were collected for each of the included studies. Any manuscripts that were proven to be assessing the same patient sample were considered a single study in order to avoid double counting of patients. Non-union and delayed union classification was determined at the original author’s discretion.

### Outcomes

The raw number of healed patients was extracted from each included study, as well as the percentage of healed patients (healing rate), for instrumented, infected, and fragility fracture non-union patients. If specific numbers for instrumented, infected, and fragility fracture non-union groups were not provided, a study was categorized in a subgroup if 80% or more of its patients were within one category.

### Data analysis

Study heal rates were analyzed as pooled proportions. Case reports were not included within the pooled analysis, as they are not capable of providing an actual incidence of healing. A simple assessment of healing rates, such as counts and percentages, were reported as the total “n” of healed patients across all studies divided by the total sample size across all studies. Pooled analysis of proportions was conducted using the OpenMeta software. All pooled proportions were reported with their associated 95% confidence intervals (CI). Analyses were conducted using a random effects model, and I^2^ was reported to summarize heterogeneity.

## Results

### Study selection

**Fig. 1:**
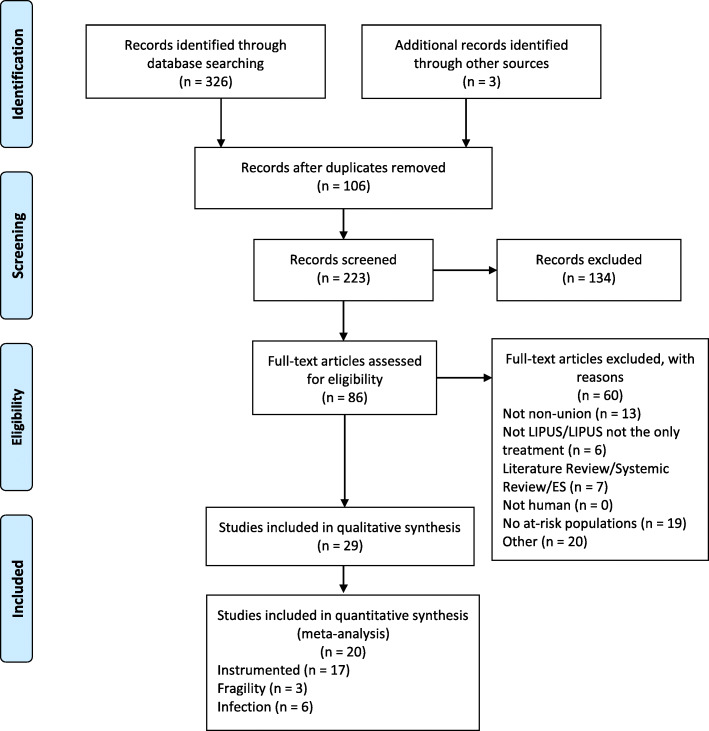
Literature review flow diagram

The literature search identified a total of 326 articles, while searching reference lists and grey literature identified an additional 3 articles. After title and abstract screening, 89 articles were eligible for full text screening. There was a total of 29 articles included in this review [9–37]. There were 20 articles included in our quantitative analysis, while the other 9 articles were case reports. There were 17 articles that provided heal rates for instrumented non-unions, 3 articles reporting fragility fracture non-unions, and 6 articles reporting on infected non-unions. A detailed summary of the article screening process is provided within Fig. 1.

### Study characteristics

The most common design of included studies was case series (17 articles), followed by case reports (9 articles). The location of research was most frequently Japan (6 studies), followed by Germany (5 studies). Studies were primarily retrospective (18 studies), with 10 prospective studies included in this review. One study combined a retrospective dataset with additional data that was captured prospectively. Studies ranged in sample size from single case reports to a series of 1359 consecutive patients, and included a wide variety of bones treated. The mean age in included studies ranged from 17 to 77, and studies included different proportions of females and males, ranging from exclusively males to exclusively females. Detailed individual study characteristics are provided in Table 1.

**Table 1:** Study Characteristics

Article First Author/Year	Country	Study Design	Retrospective or Prospective	Total Participants (n)	Mean Age (Years)	% Female	Bones Treated (Total Study)
Akiyama, 2011 [9]	Japan	Case Report	Retrospective	15	58.9	91	Femur, Hip
Exogen Post-Market Approval - EXOGEN Validation Report, 2019 [10]	USA	Case Series	Prospective	158	61	93.7	Hamate, Scaphoid, Tibia, Fibula, Ankle, Radius, Femur, Matatarsal, Clavicle, Scapula, Ulna, Humerus, Patella, Pelvis, Cuneiform, Talus
Fujishiro, 2005 [11]	Japan	Case Report	Retrospective	1	17	0	Femur
Gebauer, 2005 [12]	Germany, Austria	Case-control	Retrospective	67	46	38.8	Tibia, Fibula, Femur, Radius, Ulna Humerus, Metatarsal, Ankle, Scaphoid, Clavicle, Pelvis, Calcaneus, Rib, Knee
Hemery, 2011 [13]	France	Case Series	Retrospective	14	39	21.4	Femur, Tibia
Huber, 2012 [14]	Germany	Case Report	Retrospective	1	19	0	Phalanx
Jingushi, 2007 [15]	Japan	Case Series	Prospective	72	40.4	27.8	Humerus, Radius, Ulna, Femur, Tibia
Joshy, 2007 [16]	UK	Case Series	Retrospective	7	56	28.6	Tibia
Lee, 2016 [17]	Japan	Case Report	Retrospective	2	40	50	Femur
Lerner, 2004 [18]	Israel	Case Series	Retrospective	18	32	16.7	Femur, Tibia, Radius, Ulna, Humerus
Lim, 2012 [19]	Malaysia	Case Report	Retrospective	1	60	100	Femur
Majeed, 2019 [20]	UK	Case Series	Prospective	47	56.6	44.7	Tibia, Ankle, Foot
Mayr, 2000 [21]	USA, Germany	Case Series	Retrospective, Prospective	1359	NR	NR	Clavicle, Humerus, Radius, Ulna, Scaphoid, Femur, Tibia, Fibula, Ankle, Calcaneus, Tarsal naviculare, Metatarsal, Foot, Other
Mayr, 2002 [22]	Germany	Case Series	Prospective	100	44	37	Clavicle, Humerus, Radius, Ulna, Scaphoid, Femur, Tibia, Fibula, Other
Mirza, 2018 [23]	UK	Case Series	Retrospective	18	57.6	50	Foot, Ankle
Moghaddam, 2016 [24]	Germany	Case Series	Prospective	19	43	5.3	Humerus, Radius, Ulna, Femur, Tibia
Niikura, 2016 [25]	Japan	Case Report	Retrospective	1	44	0	Tibia
Nolte, 2001 [26]	Netherlands	Case Series	Retrospective	29	47	41.4	Tibia, Fibula, Femur, Radius, Ulna, Scaphoid, Malleolus, Clavicle, Humerus, Metatarsal
Pigozzi, 2004 [27]	Italy	Case Series	Prospective	15	35.5	20	Wrist, Scaphoid, Clavicle, Talus, Femur, Tibia, Malleolus
Povlsen, 2015 [28]	UK	Case Report	Retrospective	1	75	100	Ulna
Ricardo, 2006 [29]	Cuba	RCT	Prospective	21	26.7	0	Scaphoid
Romanò, 2006[30]	Italy	Case Series	Retrospective	49	NR	NR	NR
Roussignol, 2012 [31]	France	Case Series	Retrospective	60	43	36.7	Humerus, Ulna, Radius, Metacarpal, Femur, Tibia, Fibula, Talus, Hallux, Metatarsal, Phalanx
Rutten, 2007 [32]	Netherlands	Case Series	Prospective	71	40	21.1	Tibia
Schofer, 2010 [33]	Germany	RCT	Prospective	101	42.6	23.8	Tibia
Waseem, 2010 [34]	Canada	Case Report	Retrospective	1	77	100	Femur
Watanabe, 2013 [35]	Japan	Case Series	Retrospective	151	36.5^a^	27.2	Tibia, Fibula, Femur, Humerus, Radius, Ulna
Welch-Phillips, 2016 [36]	Ireland	Cohort	Retrospective	NR	NR	NR	NR
Zura, 2015 [37]	USA	Case Series	Prospective	767	45.8	45.4	Tibia, Femur, Radius, Ulna, Humerus, Fibula, Scaphoid, Ankle, Metatarsal, Foot

*Abbreviations*: *NR* Not Reported, *RCT* Randomized Controlled Trial

^a^Median

### Pooled healing rates

**Fig. 2:**
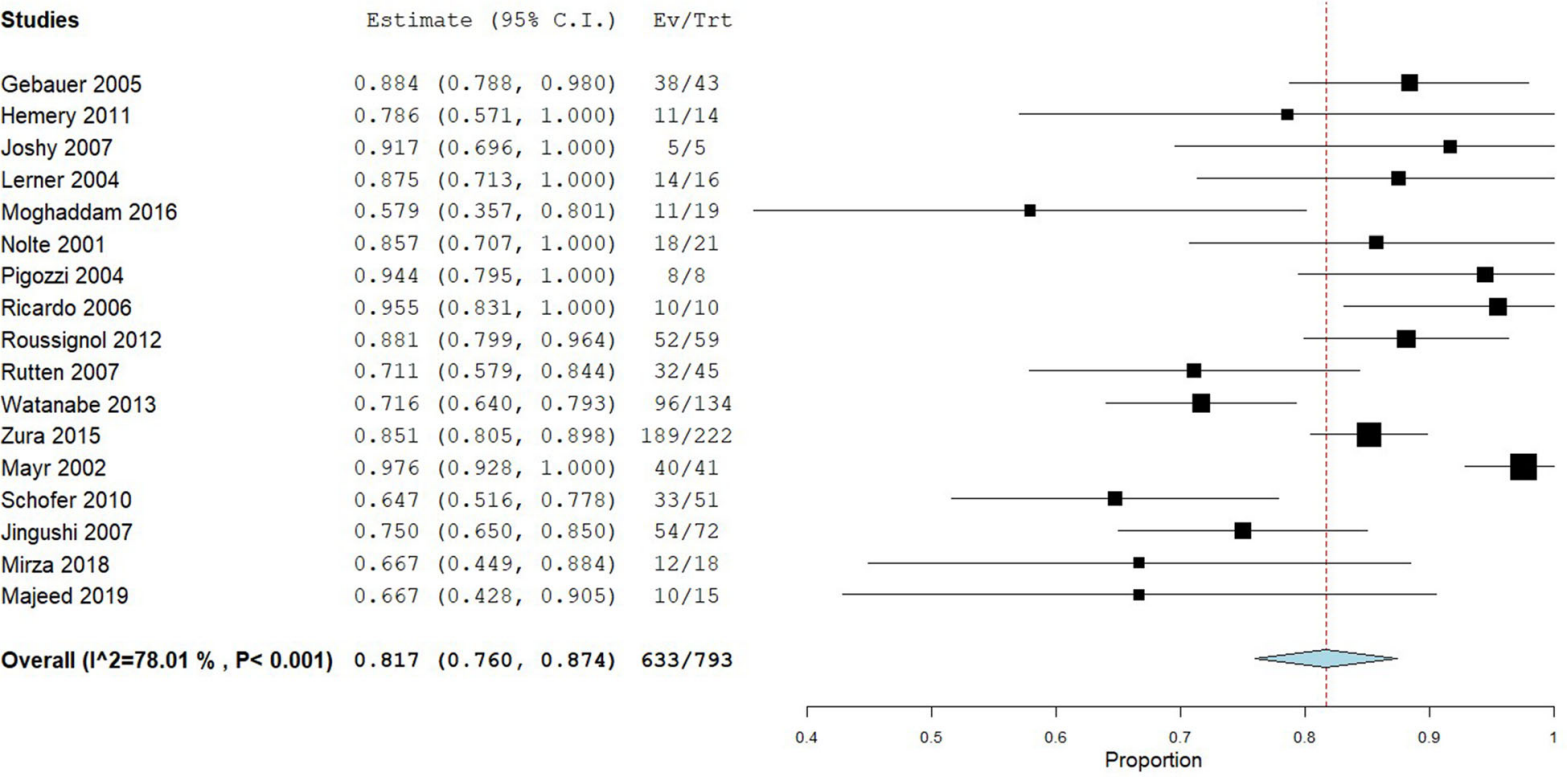
Shows the pooled proportion for instrumented non-union

**Fig. 3:**
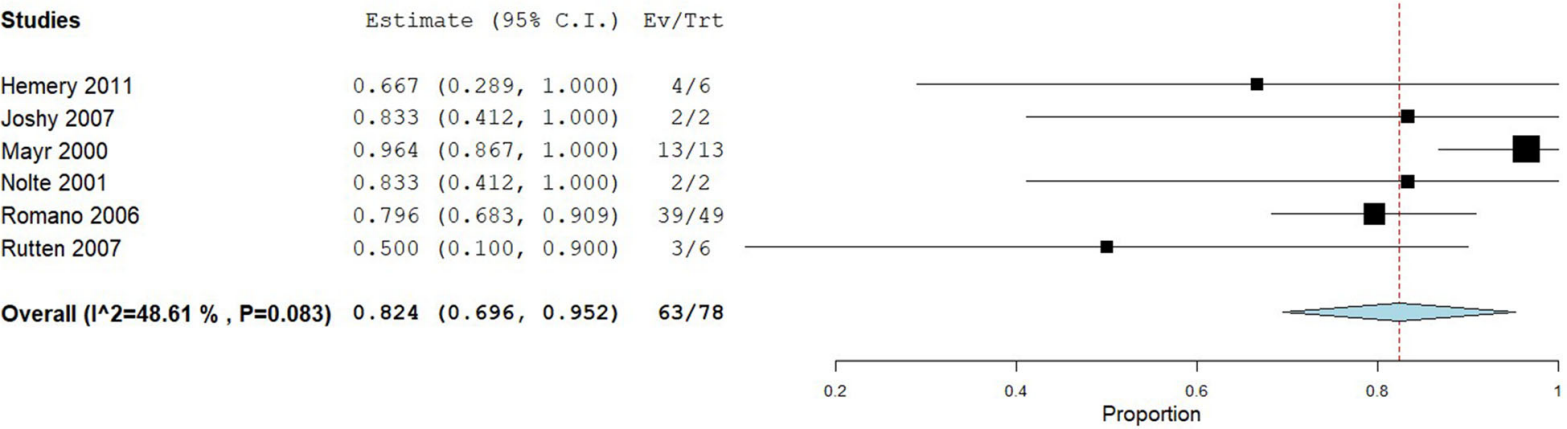
Shows the pooled proportion for infected non-union

**Fig. 4:**
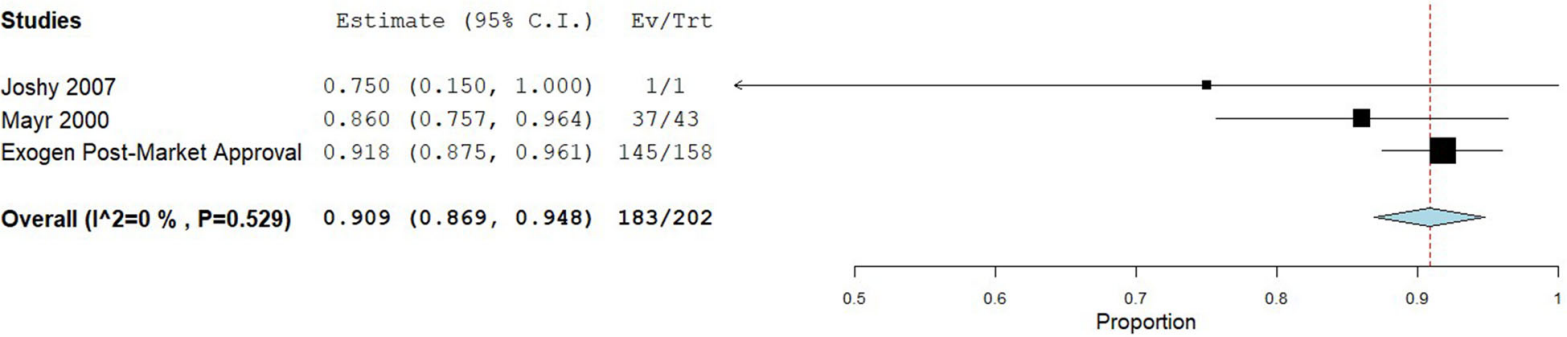
Shows the pooled proportion for fragility non-union

Healing rates within each of the included studies are provided in Table 2. A total of 793 patients were included in the analysis of healing rate for instrumented fractures, 78 patients with infected non-unions, and 202 patients with fragility fracture non-unions. Instrumented non-unions had a pooled healing rate of 82% (95% CI: 76 to 87%), with substantial heterogeneity observed (I^2^ = 78%) (Fig. 2). Infected non-unions had a similar pooled healing rate of 82% (95% CI: 70 to 95%), with moderate heterogeneity observed (I^2^ = 49%) (Fig. 3). Fragility fracture non-unions had a pooled healing rate of 91% (95% CI: 87 to 95%), with no heterogeneity observed (I^2^ = 0%) (Fig. 4).

**Table 2:** Heal Rates for At-Risk Sub-Populations (Including Case Reports)

Article First Author/Year	Delayed Union or Non-Union	Total Sub-Population Participants (n)	Sub-Population Participants Healed (n)	Sub-Population Heal Rate (%)
**Instrumented**				
Akiyama, 2011 [9]	Non-union	1	1	100
Akiyama, 2011 [9]	Delayed Union	1	1	100
Fujishiro, 2005 [11]	Non-union	1	1	100
Gebauer, 2005 [12]	Non-union	43	38	88.4
Hemery, 2011 [13]	Non-union	14	11	78.6
Huber, 2012 [14]	Non-union	1	1	100
Jingushi, 2007 [15]	Non-union	32	21	65.6
Jingushi, 2007^a^ [15]	Delayed Union	40	33	82.5
Joshy, 2007 [16]	Non-union	5	5	100
Lee, 2016 [17]	Non-union	1	1	100
Lee, 2016 [17]	Delayed Union	1	1	100
Lerner, 2004 [18]	Delayed Union	16	14	87.5
Lim, 2012 [19]	Non-union	1	1	100
Majeed, 2019 [20]	Non-union	15	10	66.7
Mayr, 2000 [21]	Delayed Union	NR	NR	91.7
Mayr, 2002 [22]	Combined Delayed Union & Non-union	41	40	97.6
Mirza, 2018 [23]	Combined Delayed Union & Non-union	18	12	66.7
Moghaddam, 2016 [24]	Non-union	19	11	57.9
Niikura, 2016 [25]	Non-union	1	1	100
Nolte, 2001 [26]	Non-union	21	18	85.7
Pigozzi, 2004 [27]	Non-union	8	8	100
Povlsen, 2015 [28]	Non-union	1	1	100
Ricardo, 2006 [29]	Non-union	10	10	100
Roussignol, 2012 [31]	Non-union	59	52	88.1
Rutten, 2007 [32]	Non-union	45	32	71.1
Schofer, 2010^a^ [33]	Delayed Union	51	33	64.7
Waseem, 2010 [34]	Non-union	2	2	100
Watanabe, 2013 [35]	Non-union	42	27	64.3
Watanabe, 2013 [35]	Delayed Union	92	69	75
Welch-Phillips, 2016 [36]	Delayed Union	NR	NR	73.5
Zura, 2015 [37]	Non-union	222	189	85.1
**Infection**				
Fujishiro, 2005 [11]	Non-union	1	1	100
Hemery, 2011 [13]	Non-union	6	4	66.7
Joshy, 2007 [16]	Non-union	2	2	100
Lim, 2012 [19]	Non-union	1	1	100
Mayr, 2000 [21]	Combined Delayed Union & Non-union	13	13	100
Niikura, 2016 [25]	Non-union	1	1	100
Nolte, 2001 [26]	Non-union	2	2	100
Romanò, 2006	Non-union	49	39	79.6
Rutten, 2007 [32]	Non-union	6	3	50
Waseem, 2010 [34]	Non-union	1	1	100
**Fragility**				
Joshy, 2007 [16]	Non-union	1	1	100
Mayr, 2000 [21]	Combined Delayed Union & Non-union	43	37	87
Exogen Post-Market Approval - EXOGEN Validation Report, 2019 [10]	Non-union	158	145	91.8

*Abbreviations*: *NR* Not Reported

^a^Overall heal rate is reported as the authors did not provide a subgroup analysis of heal rate in instrumented non-union fractures

## Discussion

The results of this study demonstrate that, given the available literature, LIPUS treatment healed 82% of instrumented non-unions, 82% of infected non-unions, and 91% of fragility fracture non-unions. These findings support the use of LIPUS for conservative non-union management within these non-union subgroups, as these healing rates are similar to those seen for LIPUS use within the general non-union literature [38]. Previous studies have suggested that Exogen non-union heal rates are 84 to 90%, with mean healing times of 178 days [6]. In comparison, surgical management of non-unions has also demonstrated comparable healing rates and comparable times to heal a non-union [6]. It is important to consider the improved socioeconomic implications of healing a non-union nonoperatively within these groups, as resorting immediately to surgical treatment may pose unnecessary costs to the health care system. It is also important to measure the quality of life implications that would be also improved with a successful non-operative LIPUS treatment protocol. With over 80% of patients demonstrating a healed non-union with LIPUS, regardless of infection, instrumentation, or fragility fracture, the costs of surgical management may be avoidable in a large proportion of non-union patients.

This strength of this investigation is its comprehensive review of the currently available literature. The systematic search and screening process provide certainty that all available literature has been documented and summarized within this study. Additionally, this study is strengthened by its applicability and direct implications for current clinical orthopedic practice. The socioeconomic costs of surgical management of non-unions can be large, and this study provides evidence that conservative non-union treatment is a viable option that would limit the exposure to these exorbitant costs.

Despite these strengths, there are a number of key limitations that must be considered when interpreting these findings. The weaknesses of this study will be detailed as follows: 1) the current available evidence is primarily from case series data. There is a lack of high quality, level 1 evidence that assesses the effect of LIPUS on non-union management. This precludes the ability to comparatively assess LIPUS with a published prospective study. Within this form of pooled proportion analysis there is no comparator, thus making all included data, including that from RCT and cohort studies, should still be considered as low quality. 2) the classification of non-union may not be the same within each of the included studies. While all studies identified their patients as having non/delayed union, they did not all use the exact same criteria to make this classification. Although this creates possible heterogeneity across studies, they all generally classified non-union in patients as a stop in biological healing progression. While criteria may have differed slightly, the findings can be reasonably attributed to non-union patients in general. 3) Another consideration with regard to the between study heterogeneity that was observed is the inclusion of all bone types in this analysis. While differences between healing rates across bones may exist, the healing rates are consistently beneficial across studies and bones, which provides justification for pooling.

It would be beneficial for future investigations to define delayed and non-unions and provide a randomized comparison between a LIPUS non-surgical treatment protocol and surgical treatment of non-unions. This would help limit the impact that prognostic variables may have on the results, as can be seen within the majority of the current non-randomized literature. Additional analyses of the direct socioeconomic cost implications of LIPUS implementation compared to surgical intervention within non-union patient groups and subgroups would also be of benefit. This would allow more clear understanding of the potential for a LIPUS non-union treatment protocol to reduce costs and reduce the impact of a non-union to the health care system, patients and their families. It is important to consider other relevant outcomes in future investigations. While reporting of adverse events is minimal with regard to LIPUS, studies comparing LIPUS to other treatment options needs to weigh the potential adverse events of other treatments relative to LIPUS. For example, surgical interventions may demonstrate successful healing rates, but the possible adverse events would also need to be taken into consideration. Compliance with LIPUS treatment protocols is also an important aspect of treatment, as a lack of compliance may result in less than optimal healing outcomes.

## Conclusion

This study has provided a thorough overview of the current literature on LIPUS treatment for instrumented, infected, and fragility non-unions. Across all current evidence, non-union healing rates for these subgroups were 82% in instrumented, 82% in infected, and 91% in fragility non-union patients. Due to the large socioeconomic and quality of life implications for surgical management of these patient subgroups, LIPUS treatment should be considered as a conservative treatment option.

## Supplementary Information

**Additional file 1.**

## Data Availability

All data analyzed during this study are included in this published article.
