# Supplementary material for: Low intensity pulsed ultrasound (LIPUS) use for the management of instrumented, infected, and fragility non-unions: a systematic review and meta-analysis of healing proportions
Source: BMC Musculoskelet Disord. 2021 Jun 11;22:532. doi: 10.1186/s12891-021-04322-5 (PMC8196464; doi:10.1186/s12891-021-04322-5)
Supplement: Supplementary file 1 — Additional file 1. [file 12891_2021_4322_MOESM1_ESM.docx]

**Appendix A: Literature Search**

| **OVID Medline** | **OVID Embase** | **CINAHL** |
| --- | --- | --- |
| 1. (nonunion or non-union or non union).mp. or delayed union or Fractures, Ununited/ 2. (Exogen or sonic accelerated fracture healing* or SAFHS or LIPUS or low-intensity puls* or low intensity puls* or pulsed low intensity ultrasound or pulsed-low intensity ultrasound or PLIUS or melmak or osteotron).mp. 3. Ultrasonic Therapy/ 4. 2 OR 3 5. 1 AND 4 6. Limit 5 to humans | 1. (nonunion or non-union or delayed union or non union).mp. 2. exp fracture nonunion/ 3. (Exogen or sonic accelerated fracture healing* or SAFHS or LIPUS or low-intensity puls* or low intensity puls* or pulsed low intensity ultrasound or pulsed-low intensity ultrasound or PLIUS or melmak or osteotron).mp. 4. ultrasound therapy/ 5. 1 OR 2 6. 3 OR 4 7. 5 AND 6 8. Limit 7 to Humans | 1. TX (Exogen or sonic accelerated fracture healing* or SAFHS or LIPUS or low-intensity puls* or low intensity puls* or pulsed low intensity ultrasound or pulsed-low intensity ultrasound or PLIUS or melmak or osteotron) 2. (MH "Ultrasonic Therapy")      1. 1 OR 2 2. TX ( nonunion or non-union or non union ) OR (MH "Fractures, Ununited") 3. 3 AND 4 4. Limit 5 to Humans |
| **Hits: 100** | **Hits: 199** | **Hits: 27** |
